# Physical exercise improves functional recovery through mitigation of autophagy, attenuation of apoptosis and enhancement of neurogenesis after MCAO in rats

**DOI:** 10.1186/1471-2202-14-46

**Published:** 2013-04-08

**Authors:** Liying Zhang, Xiquan Hu, Jing Luo, Lili Li, Xingyong Chen, Ruxun Huang, Zhong Pei

**Affiliations:** 1 Department of Rehabilitation Medicine, the Third Affiliated Hospital, Sun Yat-Sen University, Guangzhou, 510630, China; 2 Department of Neurology, the First Affiliated Hospital, Sun Yat-Sen University, Guangzhou, 510080, China

**Keywords:** Physical exercise, Autophagy, Apoptosis, IGF-1, Neurogenesis, MCAO

## Abstract

**Background:**

Physical exercise improves functional recovery after stroke through a complex mechanism that is not fully understood. Transient focal cerebral ischemia induces autophagy, apoptosis and neurogenesis in the peri-infarct region. This study is aimed to examine the effects of physical exercise on autophagy, apoptosis and neurogenesis in the peri-infarct region in a rat model of transient middle cerebral artery occlusion (MCAO).

**Results:**

We found that autophagosomes, as labeled by microtubule-associated protein 1A light chain 3-II (LC3-II), were evident in the peri-infarct region at 3 days after 90-minute MCAO. Moreover, 44.6% of LC3-positive cells were also stained with TUNEL. The number of LC3 positive cells was significantly lower in physical exercise group than in control group at 14 and 21 days after MCAO. Suppression of autophagosomes by physical exercise was positively associated with improvement of neurological function. In addition, physical exercise significantly decreased the number of TUNEL-positive cells and increased the numbers of Ki67-positive, a proliferative marker, and insulin-like growth factor-1 (IGF-1) positive cells at 7, 14, and 21 days after MCAO.

**Conclusions:**

The present results demonstrate that physical exercise enhances neurological function possibly by reduction of autophagosome accumulation, attenuation of apoptosis and enhancement of neurogenesis in the peri-infarct region after transient MCAO in rats.

## Background

Ischemic stroke is a major cause of neurological disability and a big burden on the family and society. Regaining function can significantly reduce dependence and improve the quality of life of stroke survivors. Ischemic stroke has a very complex pathophysiology. In addition to irreversible neuronal damage, ischemia also triggers cellular processes for neuronal repair involving remaining neurons. Apoptosis and necrosis are two vital types of cell death in ischemic brain injury [1]. Recently, autophagic cell death has been reported as a third type of cell death in ischemic tissue [2,3]. Autophagy is a lysosomal pathway for recycling of organelles and long-lived proteins [4,5]. In the course of autophagy, autophagosomes or autophagic vacuoles, are formed to sequester cytoplasmic constituents [6]. The autophagosomes fuse with lysosomes to digest the contents for recycling. Physiologically, autophagy plays a key role in adapting to nutritional deprivation and eliminating aggregated proteins [7]. However, inappropriate activation of autophagy may lead to cell death in cerebral ischemia [2,3,8,9]. Although it is unclear whether autophagy prevents or contributes to apoptotic cell death, the interaction between autophagy-related and apoptosis-related proteins, suggests an interplay between apoptosis and autophagy [10,11]. On the other hand, stroke also induces neurogenesis [12,13]. It has been reported that newborn neurons can contribute to functional recovery after stroke [1,12]. Interestingly, down-regulation of either autophagy or apoptosis can increase neurogenesis after stroke [1]. Therefore, the functional outcome may be resulted from a complex interplay among autophagy, apoptosis and neurogenesis following cerebral ischemia.

Previously, we and others have demonstrated that physical exercise can improve functional recovery after stroke [14]. The protective effects of physical exercise are partially associated with enhancement of neurogenesis and attenuation of apoptosis [15,16]. It is necessary to investigate the effects of physical exercise on neuronal proliferation and death. Although it has been proved that physical exercise can mitigate autophagy and enhance functional recovery after myocardial infarction in animals [17], the role of autophagy in exercise-induced functional recovery after stroke remains elusive. Growth factors such as IGF-1 also have benefitial effects on exercise-induced functional recovery in cerebral ischemia [18-20]. It is also reported that up-regulation of IGF-1 expression mitigates autophagy in some conditions [21]. Consequently, the aim of this study is to investigate the effects of physical exercise on ischemia-induced autophagy, apoptosis, neurogenesis and IGF-1 in the peri-infarct region after transient middle cerebral artery occlusion (MCAO) in rats.

In this study, we demonstrated that physical exercise could mitigate autophagosome accumulation, attenuate apoptosis, promote neurogenesis and IGF-1 expression in the peri-infarct region, thus improving the functional recovery.

## Results

### Physical exercise improved functional recovery

**Figure 1:**
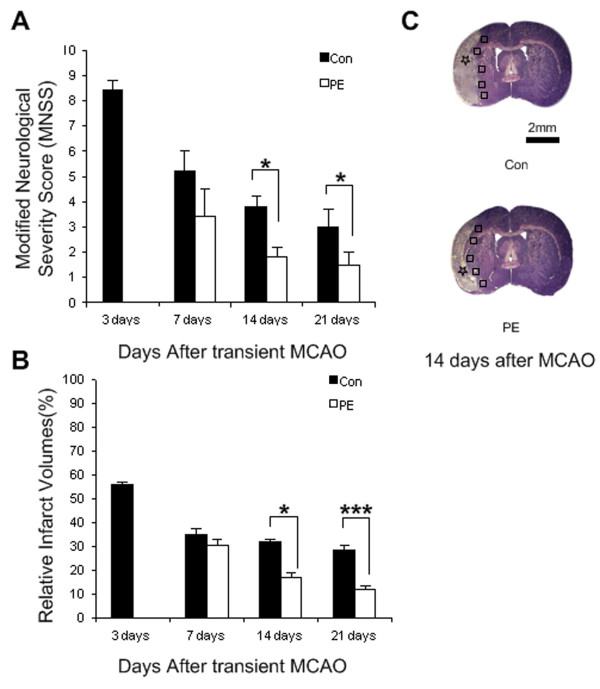
**Neurological function score, nissl staining, infarct area. (A, B)** Neurological function scores and infarct area in control and physical exercise group (n=10). **(C)** Nissl staining of brain tissues at 14 days after transient MCAO. The location of infarct area was labelled by black stars, and the peri-infarct region was labelled by black squares. Values are mean ± SD, * p< 0.05, *** p< 0.001. Scale bar in C apply to C. PE = physical exercise and Con = control.

The effects of physical exercise on neurological function were evaluated using Modified Neurological Severity Score (MNSS) scale. The MNSS were 4.2±1.1, 1.8±0.4 and 1.5±0.5 at 7, 14 and 21 days in the exercise groups after MCAO, respectively. In contrast, the MNSS were 8.4±0.4, 5.2±0.8, 3.8±0.4 and 3.0±0.7 at 3, 7, 14 and 21 days in the control group after MCAO, respectively. The repeated measures ANOVA revealed a significant main effect of MNSS (MNSS of physical exercise groups < MNSS of control groups) at 14 (F_1,8_= 8.06, p =0.022) and 21 (F_1,8_= 5.884, p =0.038) days, an significant interaction between treatment effects and time effects at 14 (F_2,16_= 8.063, p =0.004) and 21 (F_3,24_= 11.405, p < 0.001) days and a significant time effect at 14 (F_2,16_= 187.111, p < 0.001) and 21 (F_3,24_= 538.097, p < 0.001) days. But there was no significant difference between two groups at 7 days (p=0.272) after MCAO. Non-parametric analysis revealed that the MNSS values at 7, 14 or 21 days were much lower than those in control group at 3 days (p<0.001, Figure 1A), indicating a spontaneous recovery after MCAO.

There was no significant difference in mean arterial pressure, rectal temperature, arterial blood gas values, glucose levels and body weight (data not shown).

### Physical exercise reduced the volume of the infarct area

The relative infarct volumes were 57.5 %±1.1% at 3 days in basal control group after MCAO. The relative infarct volumes were 30.5%±2.3% and 35.1%±2.5% in physical exercise and control group at 7 days after MCAO. The relative infarct volumes were 16.8%±1.9% and 13.0%±1.7% at 14 and 21 days in the physical exercise group after MCAO, respectively. In contrast, the relative infarct volumes were 31.5%±1.2% and 28.9% ±1.5% at 14 and 21 days in the control group after MCAO, respectively. In control group, the infarct volumes were significantly smaller at 7, 14 and 21 days than those at 3 days after MCAO (p<0.001, Figure 1B), indicating a spontaneous recovery after MCAO. Compared with control group, physical exercise significantly reduced the infarct volumes at 14 (p<0.05) and 21 days (p<0.001) but not at 7 days (p>0.05) after MCAO (Figure 1B and C). Interestingly, the attenuation of MNSS was positively correlated with the reduction of infarct volumes (r=0.933, p<0.001). These findings suggest that physical exercise reduces brain damage and improves neurological function.

### Physical exercise mitigated autophagosomes accumulation and attenuated apoptosis in the peri-infarct region

**Figure 2:**
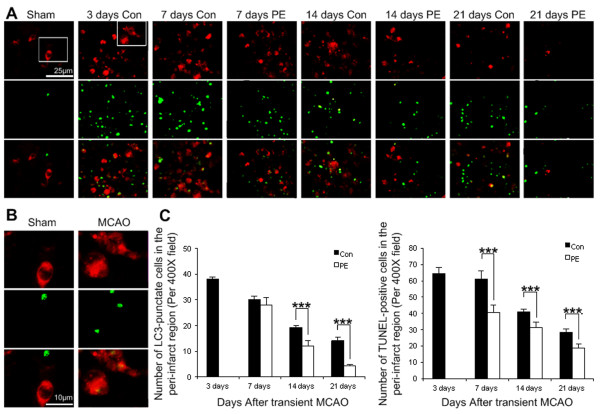
**Expression of LC3/TUNEL-positive cells in the peri-infarct region. (A, B)** LC3-punctate cells co-located with TUNEL-positive cells in the peri-infarct region. **(C)** Quantification of LC3-punctate cells and TUNEL-positive cells (n=5). Values are mean ± SD, *** p< 0.001. Scale bars in (**A, B**) apply to (**A, B**), respectively. PE = physical exercise and Con = control.

To examine the involvement of autophagosomes, immunostaining was performed using an antibody against LC3. There are two forms of LC3—the cytosolic (LC3-I) and membrane-bound (LC3-II) forms. Upon induction of autophagy, the cytosolic LC3-I is conjugated to phosphatidylethanolamine to form LC3-II and the latter then translocates to the newly formed autophagosome membrane. Therefore, LC3 staining shows a change from diffuse cytoplasmic pattern to intense punctate labelling when autophagosome formation is induced. LC3 staining remained diffuse within the cytoplasm in sham-operated rats or contra-lateral hemisphere (Figure 2B). In contrast, LC3 staining displayed numerous punctate dots within the cytoplasm after transient MCAO (Figure 2B). LC3-immunopositive cells reached the peak in the peri-infarct region at 3 days after MCAO and decreased thereafter (Figure 2A). There were significant differences in the number of LC3-punctate cells between control and physical exercise groups at 14 (p<0.001) and 21 days (p<0.001), but not at 7 days (p>0.05) (Figure 2C). Compared with control group, physical exercise alleviated autophagy. Furthermore, the number of LC3-punctate cells was positively correlated with both neurological function scores (r=0.901, p<0.001) and relative infarct volumes (r=0.832, p<0.001), suggesting that activity of autophagy is associated with ischemic cellular injury. Therefore, physical exercise improves functional recovery may, at least partially, through inhibition of autophagy.

The TUNEL-positive cells were rare in the contra-lateral hemisphere and sham-operated group after MCAO. In contrast, the TUNEL-positive cells were evident in peri-infarct region at 3 days and then decreased gradually from 7 to 21 days (Figure 2A). There were significant differences in the number of TUNEL-positive cells between physical exercise group and control group at 7, 14 and 21 days (all p<0.001), suggesting that physical exercise reduces apoptotic cell death (Figure 2C).

To further investigate the demise of LC3-positive cells, double-labeled immunofluorescence staining was performed using antibodies against LC3 and TUNEL. Notably, double staining showed that 44.6% of LC3-positive cells were also stained with TUNEL (Figure 2A and 2B). LC3/TUNEL double-positive cells were significantly lower in physical exercise groups than in control group (p<0.05, data not shown). Moreover, the induction of LC3-punctate cells was positively correlated with the number of TUNEL-positive cells (r=0.941, p<0.001).

### Physical exercise increased the expression of IGF-1 and promoted neurogenesis in the peri-infarct region

**Figure 3:**
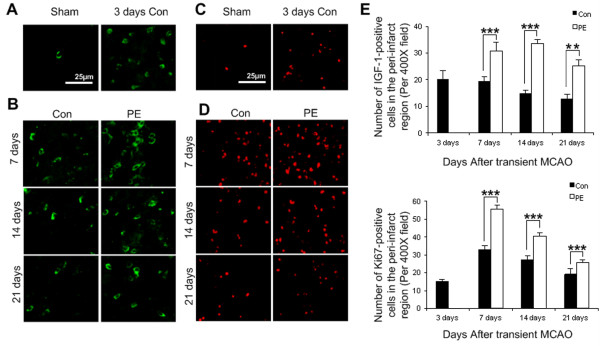
**Expression of IGF-1and Ki67-positive cells in the peri-infarct region. (A, B)** IGF-1-positive cells in the peri-infarct region. **(C, D)** Ki67-positive cells in the peri-infarct region. **(E)** Quantification of IGF-1 and Ki67-positive cells (n = 5). Values are mean ± SD, ** p< 0.01, *** p< 0.001. Scale bar in A apply to (**A, B**), and Scale bar in C apply to (**C, D**), respectively. PE = physical exercise and Con = control.

IGF-1-positive cells were evident on ischemic side but were rarely detected on the contra-lateral hemisphere (Figure 3A and 3B). There was no co-localization between IGF-1 and LC3-II. Compared with control group, physical exercise increased the expression of IGF-1 at all the observed time points (p<0.001) (Figure 3E).

Ki67, a proliferative marker, was used to evaluate neurogenesis in the peri-infarct region. At 3 days after MCAO, Ki67-positive cells were obvious in the peri-infarct region, but were barely visible in the contra-lateral cerebrum and sham-operated group (Figure 3C and 3D). The number of Ki67-immunopositive cells in the peri-infarct region reached the peak at 7 days and decreased thereafter. Physical exercise significantly increased Ki67-immunopositive cells in the peri-infarct region at 7 (p<0.001), 14 (p<0.001) and 21 days (p<0.01) after MCAO (Figure 3E).

## Discussion

In the present study, we investigated the effects of physical exercise on autophagy, apoptosis and neurogenesis. We found that ischemia-induced autophagy was associated with apoptotic cell death but not with neurogenesis, suggesting a deleterious role of autophagy in brain ischemic injury. In addition to attenuating autophagy and apoptotic cell death, physical exercise also promoted IGF-1 expression and cell proliferation, thereby improving functional recovery.

The function of autophagy in exercise-mediated protection against ischemia still remains controversial. For example, in myocardial infarction, prior exercise maintains basal autophagy and protects against cardiac ischemic injury [22]. On the other hand, post-ischemic exercise reduces the ratio of LC3II/LC3I and improves functional recovery [17]. It is generally believed that imbalance or excessive autophagy promotes cellular pathology and ultimately leads to cell death in cerebral ischemia [1,23-26]. However, the relationship between autophagy and exercise-mediated neuroprotection is not clear. Consistently, we found that autophagosomes were accumulated after focal cerebral ischemia. In contrast, physical exercise attenuated ischemia-induced autophagosome accumulation (Figure 2C). The suppression of autophagy by exercise was positively associated with the recovery of neurological function (r=0.901, p<0.001) and reduction of brain damage (r=0.832, p<0.001), suggesting that physical exercise may improve functional recovery from ischemic stroke, at least in part, through inhibition of autophagy. Cell death, especially apoptosis, is a major contributor to neuronal damage in cerebral ischemia [1,11]. The interplay between autophagy and apoptosis is very complex. Autophagy can either promote or inhibit apoptosis under different conditions [11]. Interestingly, we found that TUNEL-positive and LC3-punctate cells were pronounced in the peri-infarct region and the number of TUNEL-positive cells was positively correlated with the number of LC3-punctate cells (r=0.941, p<0.001). Physical exercise significantly reduced both autophagic and apoptotic cell death. Given that autophagy and apoptosis can share many common death pathways [11]. Our findings suggest that autophagy may play a pathologic role in ischemic cell death whereas physical exercise may attenuate ischemia-induced brain damage through inhibition of common upstream signals for cell death.

Neurogenesis, particularly IGF-1-mediated neurogenesis, is a major mechanism underlying beneficial effects of exercise on ischemic stroke [18-20]. Post-ischemic neurogenesis is a complex process involving coordination of multiple signaling pathways. It has been reported that autophagy can play opposite roles in regulating neurogenesis depending on different conditions. For example, activation of autophagy can modulate cell proliferation during neuronal development and regeneration [21,27]. On the other hand, reducing autophagic activity has been shown to promote neurogenesis after ischemic stroke [1]. In addition, IGF-1 is also thought to have significant inhibitory actions on autophagy through activation of mammalian target of rapamycin complex 1 (mTORC1) [21]. Consistently, exercise significantly increased the numbers of IGF-1- positive cells and Ki67-labeled proliferation cells. However, LC3 was not co-localized with either IGF-1 or Ki-67, indicating that autophagy may not be directly involved in ischemia-induced neurogenesis. Whether IGF-1 plays a role in exercise-induced mitigation of atuophagy needs further investigation.

## Conclusions

Altogether, our results suggest that the benefit of physical exercise on functional recovery is associated with mitigation of autophagy, attenuation of apoptosis and enhancement of neurogenesis as evidenced by IGF-1 expression and cell proliferation in the peri-infarct region after stroke.

## Methods

### Animals and treatment

All experimental procedures were approved by the Institutional Animal Ethical Committee Sun Yat-sen University and were conducted according to the Guide for the Care and Use of Laboratory Animal of the National Institute of Health (Publication No. 80–23, revised 1996). A total of 40 male Sprague–Dawley rats weighing 250-280g were used for this experiment. Rats were housed in the same animal care facility during a 12 hour light/dark cycle throughout the protocol. Sprague–Dawley rats were subjected to transient focal cerebral ischemia induced by left transient middle cerebral artery occlusion (MCAO) as mentioned earlier [1,28]. Briefly, rats were anesthetized with intra-peritoneal injection of 3.5% chloral hydrate (350 mg/kg) and were placed in a supine position. Body temperature of the rats was maintained at 37±0.5°C on a heating pad. The left common carotid artery (CCA), internal carotid artery (ICA), and external carotid artery (ECA) were surgically exposed. The CCA was ligated distally and the ECA was ligated proximally to the bifurcation of the ICA and the ECA. A 3–0 silk suture was tied loosely around the ICA, and a micro-vascular clip was placed distally across the ICA. A filament (4–0 nylon suture with rounded tip) was inserted into the ICA through the CCA and gently advanced from the common carotid artery bifurcation to block the middle cerebral artery (MCA) at its origin. The suture around the ICA was tightened, and the microvasculature clip was removed. Mean arterial blood pressure, heart rate and arterial blood gases were analyzed during the process of surgery. The suture was pulled back until the tip reached the suture around the ICA to restore blood flow (reperfusion) after 90 min of MCAO. The animals were not allowed to recover from anesthesia until the wound was closed, and they were sent to their cages.

The neurological function was evaluated using the Bederson’s neurological function test, after 6 hour MCAO. The Bederson’s scores are as follow: no deficits score, 0; unable to extend the contra-lateral forelimb score, 1; flexion of contra-lateral forelimb score, 2; mild circling to the contra-lateral side score, 3; severe circling and allying to the contra-lateral side score, 4. The rats with scores 1–3 were then selected and randomly divided into three groups: the physical exercise group (n=15), which was given running exercise everyday at 3 days after transient MCAO, the control group (n=20), and sham-operated group (n=5, filament was not inserted into the artery), which were fed in standard cages with no special exercise training and served as controls. To normalize for handling stress, sedentary animals in control and sham-operated group were placed on nonmoving wheels for time duration equal to exercised treatments. Exercised rats were further randomized into one of three groups with different exercise durations: 7, 14 and 21 days groups after transient MCAO. Correspondingly, sedentary rats were randomly divided into four groups: 3 days group (basal control group) and 7, 14 and 21 days after transient MCAO.

### Exercise training and function testing

All animals submitted to the running wheel exercise were placed into a programmable, motorized wheel apparatus (21cm diameter, 40cm long, made in China), which was easy to quantify the exercise intensity. The rats in physical exercise group were put into the wheel to run at 3 days post-ischemia. At the beginning, the running speed was set as 5 rev/min (about 3m/min), for 20 minutes twice a day (morning and afternoon), then gradually increased to 10rev/min (about 6m/min) on the seventh day, 15 rev/min (about 10m/min) on the fourteenth day. The control group and sham-operated group were housed in a standard cage (n=5) with no special exercise training, supplied with enough food and water. Body weight was monitored every 3 days.

All rats in this study were given 1 week pre-conditioning exercise before MCAO and the investigator was blinded to the experimental groups. Neurological function was assessed on a scale of 0–18 (normal score, 0; maximal deficit score, 18) [14,29,30]. Neurological severity score is a combination of motor, sensory, reflex and balance tests [14,31].

### Tissue preparation for histochemistry

Rats were sacrificed after the completion of motor functional evaluation at 3, 7, 14 and 21 days after transient MCAO (n=5 per group at each time point) with an overdose of 10% chloral hydrate and perfused transcardially with 0.9% saline at 4°C followed by 4% paraformaldehyde in phosphate buffer (0.1 mol/L, pH 7.4) [14]. The brains were removed, fixed in the above fixative for 8 hours at 4°C, and then immersed sequentially in 20% and 30% sucrose until sinking occurred. Coronal sections (10-μm thick) were cut on a cryostat (CM1900; Leica, Heidelberger, Germany) from bregma +4.0 to −6.0 mm and used for Nissl staining, immunoflourescence staining or TUNEL staining.

### Nissl staining

Serial sections from bregma +4.0 to −6.0 mm were selected for Nissl staining to measure infarct volume in the ipsilateral hemisphere. Nissl staining was performed with 0.1% cresyl violet (Sigma) on basis of a standard procedure. For quantification of infarct volume, five successive coronal sections at 2.0-mm intervals from bregma +4.0 to −6.0 mm were selected. The infarct areas marked by black stars (see Figure 1C) are referred to as zones of irreversible ischemic damage that have been exposed to the most severe reduction in cerebral blood flow and exhibit severe, consistent, ischemic damage. The area immediately outside this zone is referred to as the ‘peri-infarct’, in which most neurons do not display the histological signs of irreversible damage [32]. The volumes of the ipsilateral and the contra-lateral hemisphere were counted as previously mentioned and relative infarct volume was described as a percentage of the contra-lateral hemisphere [33,34].

### Immunofluorescence staining

Another set of sections was used for immunofluorescence. Immunofluorescence staining of LC3-II, IGF-1 and Ki67 were performed as following: Sections were pretreated for 5 minutes with hot (85°C) citrate buffer (0.01 mol/L, pH 6.0) for antigen retrieval followed by 5% normal goat serum for 1 hour at room temperature. Next, sections were incubated with mouse anti-LC3 (microtubule-associated protein 1A light chain 3, 1:200; MBL, Naka-Ku Nagoya, Japan), anti-IGF-1 (insulin-like growth factor, 1:100; Millipore, USA) or rabbit anti-Ki67 (rabbit monoclonal to Ki67, 1:400; Abcam, England) overnight at 4°C. After rinsing in phosphate-buffered saline (PBS) 3 times for 5 minutes each, sections were incubated with peroxidase-marked mouse secondary antibody (anti-mouse IgG, 1:1000, Cell Signaling) or rabbit secondary antibody (anti-rabbit IgG, 1:1000, Cell Signaling) for 1 hour at room temperature. Fluorescence signals were detected with a microscope (BX51; Olympus). Negative control sections were incubated with PBS instead of primary antibodies and showed no positive signals.

### Double-immunofluorescence analysis

Double-Immunofluorescence studies were performed for LC3 plus TUNEL (the terminal deoxynucleotidyl transferase-mediated dUTP in situ nick-end labeling). Staining steps were the same as those described above: sections were pretreated for 5 minutes with hot (85°C) citrate buffer (0.01 mol/L, pH 6.0) for antigen retrieval followed by 5% normal goat serum for 1 hour at room temperature. Then, sections were incubated with mouse anti-LC3 (microtubule-associated protein 1A light chain 3, 1:200; MBL, Naka-Ku Nagoya, Japan), overnight at 4°C. After rinsing in phosphate-buffered saline (PBS) 3 times for 5 minutes each, sections were incubated with peroxidase-marked mouse secondary antibody (anti-mouse IgG, 1:1000, Cell Signaling) plus Terminal deoxynucleotidyl transferase and digoxigenin-labeled nucleotides (In Situ Cell Death Detection Kit, AP, Roche Corp., Switzerland) for 1 h at 37°C. After rinsing, the co-location of LC3 and TUNEL signals were observed with a microscope (BX51; Olympus).

### Image analysis and quantification

All histological images were captured at the same exposure and analyzed with Image-Pro Plus image analysis software (Media Cybernetics, Silver Spring, MD, USA) by one author who was not aware of the assignment of animals’ group assignment. The regions of interest were defined as a zone with 700 μm width and length in the peri-infarct region, which is immediately outside the infarct zone (Figure 1C) [1]. For cell counting of LC3/TUNEL/IGF-1/Ki67-immunopositive cells, eight consecutive sections at 240-μm intervals from bregma 0.20 to −2.20 mm were analyzed. The number of LC3/TUNEL/IGF-1/Ki67-immunopositive cells in the peri-infarct region (Figure 1C, marked by black squares) was counted by Image-Pro Plus image analysis software in 4 non-overlapping fields (425 μm X 320 μm) under X 400 magnification and was presented as the average cell number per field on each section [34]. The final cell number per rat was the average cell number of all the sections [34].

### Statistical analysis

Numerical data were presented as mean ± SD. Repeated measures ANOVA was used to evaluate MNSS variables. A nonparametric test was used to evaluate MNSS and infarct volume values. Two independent samples t-test was used for 2-group comparisons of the number of LC3/TUNEL/Ki67/IGF-1 positive cells variables. Pearson bivariate correlation was used to run correlation analysis. Statistical analysis was performed using SPSS 16.0 for windows (SPSS Inc, Chicago, IL, USA). *p < 0.05, **p < 0.01, ***p < 0.001 when comparison was made.

## Abbreviations

MCAO: Middle cerebral artery occlusion; IF: Immunofluorescence; IGF-1: Insulin-like growth factor-1; LC3-II: Microtubule-associated protein 1A light chain 3-II; AVs: Autophagic vacuoles; MNSS: Modified Neurological Severity Score; CCA: Common carotid artery; ICA: Internal carotid artery; ECA: External carotid artery; TUNEL: Deoxynucleotidyl transferase-mediated dUTP in situ nick-end labeling.

## Competing interests

The authors declare that they have no conflict of interest.

## Authors' contributions

LZ, LL, XC carried out the experiments. JL performed the statistical analysis. LZ drafted the manuscript. XH, RH participated in the design of the study and ZP conceived of the study and participated in its design and coordination and helped to draft the manuscript. All authors read and approved the final manuscript.
